# Acceptability of financial incentives for health behaviour change to public health policymakers: a qualitative study

**DOI:** 10.1186/s12889-016-3646-0

**Published:** 2016-09-15

**Authors:** Emma L. Giles, Falko F. Sniehotta, Elaine McColl, Jean Adams

**Affiliations:** 1 Institute of Health & Society, Newcastle University, Newcastle upon Tyne, UK; 2 School of Health & Social Care, Health & Social Care Institute, University of Teesside, Middlesbrough, UK; 3 MRC Epidemiology Unit, University of Cambridge, Cambridge, UK

**Keywords:** Motivation, Administrative personnel, Health behaviour, Qualitative research

## Abstract

**Background:**

Providing financial incentives contingent on healthy behaviours is one way to encourage healthy behaviours. However, there remains substantial concerns with the acceptability of health promoting financial incentives (HPFI). Previous research has studied acceptability of HPFI to the public, recipients and practitioners. We are not aware of any previous work that has focused particularly on the views of public health policymakers.

Our aim was to explore the views of public health policymakers on whether or not HPFI are acceptable; and what, if anything, could be done to maximise acceptability of HPFI.

**Methods:**

We recruited 21 local, regional and national policymakers working in England via gatekeepers and snowballing. We conducted semi-structured in-depth interviews with participants exploring experiences of, and attitudes towards, HPFI. We analysed data using the Framework approach.

**Results:**

Public health policymakers working in England acknowledged that HPFI could be a useful behaviour change tool, but were not overwhelmingly supportive of them. In particular, they raised concerns about effectiveness and cost-effectiveness, potential ‘gaming’, and whether or not HPFI address the underlying causes of unhealthy behaviours. Shopping voucher rewards, of smaller value, targeted at deprived groups were particularly acceptable to policymakers. Participants were particularly concerned about the response of other stakeholders to HPFI – including the public, potential recipients, politicians and the media.

Overall, the interviews reflected three tensions. Firstly, a tension between wanting to trust individuals and promote responsibility; and distrust around the potential for ‘gaming the system’. Secondly, a tension between participants’ own views about HPFI; and their concerns about the possible views of other stakeholders. Thirdly, a tension between participants’ personal distaste of HPFI; and their professional view that they could be a valuable behaviour change tool.

**Conclusions:**

There are aspects of design that influence acceptability of financial incentive interventions to public health policymakers. However, it is not clear that even interventions designed to maximise acceptability would be acceptable enough to be recommended for implementation. Further work may be required to help policymakers understand the potential responses of other stakeholder groups to financial incentive interventions.

## Background

Engaging in health promoting behaviours helps reduce morbidity and mortality with subsequent social, healthcare and economic benefits. Despite ongoing efforts to encourage uptake of healthy behaviours, unhealthy behaviours remain common worldwide [1]. Providing financial incentives contingent on healthy behaviours is one method to encourage these behaviours. Health promoting financial incentives (HPFI) have been defined as cash or cash-like rewards or penalties provided directly to individuals contingent on their performance of healthy behaviours [2].

A number of systematic, and other, reviews support the use of HPFI [3–11]. Non-systematic reviews have reported that HPFI are more effective for ‘one off’ behaviours such as attending for screening and vaccination, than more complex behaviours such as smoking cessation [5, 7]. However, this is not confirmed in systematic reviews. Systematic reviews find that the effects of HPFI do not vary according to incentive value or target behaviour, but may be larger in more deprived groups [3, 4]. Whilst these systematic reviews find prolonged effects of continuing incentives, effects after intervention removal appear to decrease over time – although not necessarily to extinction [3, 4, 11].

Despite this evidence of effect, the acceptability of HPFI has been questioned and they have been criticized as unethical, unfair and socially divisive [12, 13]. Acceptability of public health interventions can be considered from the point of view of a number of stakeholders. In the context of HPFI, these include policymakers responsible for intervention development, those responsible for intervention delivery, the public who may finance interventions through taxation, and potential recipients. All of these groups must be willing and able to engage with HPFI if they are to be widely implemented and their potential as behaviour change interventions exploited [14].

In a recent systematic review on the acceptability of HPFI [15], 22 empirical studies were identified. Most of these (17 of 22) were conducted with members of the public. Four studies in the review captured the views of clinicians and other practitioners working with those who received incentives [16–19]. These studies show some belief that HPFI can be effective, but also highlighted concerns around the ethics of offering rewards – although the specifics of these are not well described. Whilst one study included a small number of policymakers within their sample (*n* = 3 out of 30) [19], we are not aware of any study that has specifically focused on the views of public health policymakers and decision-makers towards HPFI.

The views of public health policymakers and decision-makers on HPFI may be particularly important as these individuals are likely to play key roles in recommending, or not, HPFI at a national level, and commissioning such interventions at a local level. Understanding their views on whether or not HPFI are appropriate interventions, and barriers and facilitators to implementation, is important for developing strategies to maximise the potential of HPFI.

The aim of this research was, therefore, to explore the views of public health policymakers and decision-makers working in England, on whether or not HPFI interventions are acceptable; and what, if anything, could be done to maximise acceptability of HPFI.

## Methods

We conducted a qualitative interview study with public health policymakers and decision-makers (referred to as ‘policymakers’ throughout) working in England. Ethical approval was provided by Newcastle University’s Faculty of Medical Sciences Ethics Committee (Approval Number: 00864; May 2015). We did not collect consent to share data widely and data will not be made available. The paper is reported in accordance with the Consolidated Criteria for Reporting Qualitative Research [20].

### Participants

We recruited individuals working in positions where they could influence and make decisions concerning the commissioning or strategic direction of local, regional and national public health improvement services in England. Sampling was purposive and we aimed to recruit at least six individuals working at each of the local, regional and national levels. We focused on England, as public health services are organised differently in other parts of the UK.

### Recruitment

Participants were identified through key informants and via ‘snowballing’ – that is, asking recruited participants to suggest others who met the inclusion criteria and might be interested in taking part in the research. We purposively selected additional participants from amongst those suggested via ‘snowballing’ to achieve our intended sample mix.

Potential participants were contacted by letter or email to introduce the study and provide a participant information sheet. Follow up phone calls allowed potential participants to ask questions and make arrangements for interviews. Letters or emails were sent confirming interview appointments (and providing a further copy of the participant information sheet) one week prior to interviews, with reminder phone calls, or emails, the day before. Participants were offered a £20 high street shopping voucher as a ‘thank you’ for taking part.

### Data collection

ELG conducted interviews in person (*n* = 1) or by telephone (*n* = 20) according to participant preference. Participants took part in one interview each during working hours. Only the participant and interviewer were present during interviews.

Before interviews began, the researcher asked participants to confirm that they had read the information sheet and if they had any questions. The researcher then asked participants to complete a written consent form and (during telephone interviews) return this via email.

A semi-structured topic guide shaped interviews (Appendix 1). This was iteratively refined during interviews to improve question ordering and flow. We sent participants a series of show cards to be used during interviews (Appendix 2) via email one week before interviews.

Interviews began with general questions concerning the participant’s professional role. Then the researcher introduced the concept of HPFI, read a definition of HPFI developed from the peer-reviewed literature [2] from a show card, and asked the participant to provide their general responses. Next, the researcher read summary information from a recent systematic review [4] on the effectiveness of HPFI from a show card and asked participants if they had any specific responses to this ‘evidence’. Next, the researcher read out three examples of HPFI schemes from show cards and asked participants about the barriers and facilitators to introducing such schemes - both in general and from the specific perspective of their current position. All of the example schemes were based on, or adapted from, real scenarios [21–23] and were selected to cover a range of different behaviours and HPFI formats. Finally, the researcher summarised a framework [2] describing different aspects of HPFI design (from a show card) and asked participants how these aspects of design influenced acceptability.

At the end of interviews, the researcher summarised the key points covered and offered participants the chance to add to, revise or clarify their views. Transcripts were not returned to participants for checking and they were not asked to provide feedback on the results.

### Data analysis

With consent, all interviews were audio recorded and transcribed verbatim for analysis alongside any interviewer reflections. We used Framework Analysis [24] to analyse transcripts. We developed an initial framework based on preliminary analyses of concepts from interviews and the results of our previous work on acceptability of HPFI [14, 15, 25–27]. We then applied this to the data to identify and code pertinent extracts. Extracts that reflected concepts insufficiently identified by the framework were used to modify the framework. Thus, we iteratively refined the framework until we had a definitive version that captured all concepts and offered a coherent, structured, and cohesive account of stakeholders’ views.

The first author (ELG) conducted coding using NVivo software. Frequent discussions with the project lead (JA), ensured that data interpretation was credible, valid and shared [28].

### Reflexivity

ELG is an experience qualitative researcher [13, 27, 29–31] with a PhD in public health research. At the time interviews were conducted, she was working as a research associate, and then senior lecturer, in public health. The research was the final part of a four year programme of work on HPFI that ELG was employed on. Thus, ELG had an in-depth knowledge of HPFI. ELG had previously established professional relationships with some, but not all, of the participants before interviews were conducted.

## Results

Twenty two individuals were invited to take part, and 21 interviews were conducted during May-July 2015. One invitee refused to take part as they had retired. Interviews lasted for 20–47 min. Five participants were working at national, 10 at regional, and six at local level (see Table 1). Nine participants were male. Eight participants worked in a commissioning role, with some commissioning financial incentives, and others non-financial incentives (or a combination). Participants’ portfolios covered a range of public health functions and areas.

**Table 1:** Participant characteristics

Participant ID	Geographical level of current position	Current portfolio	Currently employed in a commissioning role
1	Regional	Smoking cessation	No
2	Local	Public health	Yes
3	Regional	Public health	Yes
4	Regional	Alcohol	No
5	Regional	Smoking cessation	No
6	Regional	Health protection	No
7	National	Public health	Yes
8	Local	Public health	Yes
9	Regional	Public policy	No
10	National	Public health	Yes
11	Regional	Drugs and alcohol	No
12	Local	Sexual health	Yes
13	Local	Sexual health	Yes
14	Local	Substance misuse	Yes
15	Regional	Mental health	No
16	National	Health and wellbeing	No
17	Local	Public health	No
18	National	Unknown	Unknown
19	Regional	Health improvement	No
20	Regional	Public health	No
21	National	Public health	No

The final coding framework is described in Table 2. The results are described, and illustrated using verbatim quotes, according to the two main research questions – factors influencing overall acceptability of HPFI; and what, if anything, could be done to maximise acceptability. Methods of maximising acceptability were primarily related to format and design of HPFI schemes and these are described with reference to a previously described framework [2].

**Table 2:** Coding tree

Code	Description
Potential benefits	
Initial motivation	HPFI generate initial motivation for healthy behaviours
Practical considerations	
Effectiveness	Considerations around initial and long-term effectiveness
Cost-effectiveness	Considerations around value-for-money
Monitoring	Considerations around ‘gaming’ and how this can be avoided
Intervention paradigm	HPFI do not address the ‘root causes’ of unhealthy behaviours
Views of others	Considerations around how other stakeholders may view HPFI
Ethical considerations	
Culture of entitlement	HPFI create a culture of entitlement
Discrimination	HPFI are discriminatory and divisive
Incentive design format	
Direction	A positive reward or negative penalty
Form	Cash, vouchers, or specific goods and services
Certainty	Certain, uncertain chance, or certain chance
Magnitude	Total value of the incentive
Recipient	Individual, group, significant other, clinician or parent
‘Other’ issues	
Free coding…	…

Despite the concerns and issues described below, participants acknowledged that HPFI could be useful interventions. It was recognised that HPFI could be a “hook” for encouraging people to adopt healthy behaviours; that HPFI could help more than the individual who receives the incentive (e.g. unborn children, if HPFI are targeted at pregnant women); and that they can help to create a culture where healthy behaviours become the norm.

*“So the micro, yes it will be better to the individual child, absolutely, and the macro is if that small trial in turn triggers community changes of behaviour…”* [ID: 21]

### Factors influencing HPFI acceptability

#### Effectiveness and cost-effectiveness

Many participants discussed the need for robust evidence on the effectiveness and cost-effectiveness of HPFI schemes. The implication being that HPFI could be acceptable if they were demonstrably effective and cost-effective. Evidence requirements for demonstrating effectiveness were high with, for example, a demand for evidence of effects sustained beyond 12 months follow up and discussion about the potential selection bias of existing studies. It is not clear if this standard of evidence is required for all potential interventions, or if this was driven by an underlying cautiousness about HPFI in particular. The systematic review evidence presented in the show card did not appear to change many views towards HPFI.

*“I think we’ll be much more open as a Public Health community to using incentives, but at the moment most of what I’ve seen has been maximum of kind of a year follow-up.”* [ID:16]

*“Well I think inevitably and absolutely unavoidably there is a selection bias in the people who participate in these studies … I think evidence on individual level behaviour change of any sort is making a biased comparison.”* [ID:18]

The current context of public sector austerity in England appeared to drive a particular interest in cost-effective, and even cost-saving, public health interventions.

*“I think the, you know, obviously the biggest factor is the question of how effective they are and whether they are cost-effective* [and] *cost-saving…”* [ID: 02]

*“It’s much easier to make an argument, as I was saying earlier, where you can demonstrate a cost-saving element to what’s being done rather than an additional cost in order to encourage the behaviour.”* [ID: 02]

#### Monitoring and avoidance of ‘gaming’

Participants raised concerns about ‘gaming the system’ – where individuals lie about their behaviour in order to gain rewards that they are not entitled to. This led to discussions about whether health behaviours could ever be monitored well enough to ensure that all gaming was identified. There appeared to be concerns that monitoring and avoiding gaming would place such heavy demands on schemes that they would become unworkable.

*“I think it’s any scheme is open to misuse and I think you will always get the edited version from somebody and some of it is the hard measured stuff is obviously far more robust.”* [ID:13]

#### Intervention paradigm

Some participants felt that HPFI failed to address the root causes of unhealthy behaviours. In particular, HPFI were identified as individual-level (rather than population-level) interventions that fail to change the context in which behaviours are performed. For this reason, HPFI were identified as a “sticking plaster”, rather than a longer term solution. Thus HPFI were identified as reinforcing a flawed focus on individual, rather than environmental and social, determinants of health behaviours.

*“I think they may have a part to play but I’m very concerned that the vast majority of the activities that we see taking place in relation to lifestyle behaviours are focused on trying to change the behaviour of individuals rather than trying to change the environment.”* [ID:18]

*“… you’d like to think that adults could be better educated earlier on in say the schooling years to become aware of healthier options, healthier choices. And that would in my view be a more effective, more strategic approach to the problem than the short-term sticking plaster* [of incentives]*.”* [ID:21]

#### Views of other stakeholders

Participants were often concerned about how other key stakeholders would perceive HPFI. These included: elected politicians, the wider public, recipients of HPFI, and the media. There was a strong feeling that all of these stakeholders would have to find HPFI acceptable before they could be implemented.

*“…we obviously need the buy-in of our partner organisations, of our commission services … making sure that the* [elected, local] *Councillors are on board, that you’re not going to get a negative public backlash, things like that are a bit more secondary but the buy-in is the crucial one.”* [ID:08]

*“And again, you know, if you get into those large types of things then you’re going to have a lot more political wrangling and you know, perhaps negative press and things, so you have to be very careful with it.”* [ID:19]

Some responses in relation to more versus less acceptable formats of HPFI (described below) appeared to relate to perceptions of what would be most acceptable to other stakeholders. For example, whilst participants believed that recipients of HPFI would value cash more than voucher rewards, vouchers were perceived to be more acceptable to other stakeholders and so preferable. Similarly, whilst participants acknowledged that recipients might prefer higher value rewards, these were felt to be less acceptable to other stakeholders and, hence, smaller value rewards were preferable overall.

*“Again, it’s that balance, isn’t it, what’s the value to the client to make it worthwhile? So, again, that would be quite interesting to look at. Are they more likely to engage in positive behaviour if it’s £5 or £10 or whatever? What size incentive is necessary and I guess we don’t know that really.”* [ID:03]

#### Ethical concerns

Irrespective of whether HPFI were effective and cost-effective, many participants felt uneasy with the approach for moral and ethical reasons. Two key concerns were highlighted – that HPFI may generate a “culture of entitlement” encouraging a belief that healthy behaviours should be instantly rewarding, and that HPFI discriminate against those who already pursue healthy behaviours.

*“I think there’s a long term risk that you’re generating an instant reward culture for behaviour change which is quite dangerous actually.”* [ID:16]

*“… it’s not ethical, you know, I’ve got them, you know, actually we’ve got lots of people who are already engaged in healthy behaviours and so why should they not be able to access some incentive for* [that] *already?”* [ID:17]

### Maximising acceptability

#### Direction of incentive – rewards versus penalties and deposit schemes

The majority of participants thought that reward-based incentives were more acceptable than penalties or deposit schemes. This was mainly because they felt rewards provided a positive recognition of the effort made by individuals attempting behaviour change.

*“I would always favour rewards as opposed to penalties… It’s reinforcing the positivity of the intervention.”* [ID:03]

Deposit schemes were viewed unfavourably by most participants as they were perceived as excluding large groups who did not have money to spare – often the very groups felt to be most in need of help to improve their health.

*“I think that there’s something very odd about requiring the individual to deposit a lump sum at the beginning … it would immediately exclude a pretty large part of the population we wanted to try and get it to, because they simply wouldn’t have the money to do that.”* [ID:02]

*“My initial response was ‘gosh, how middle class’, you know? I can’t imagine any of the deprived communities that I have worked with directly being able to deposit a lump sum like that into a scheme and to risk not getting it back.”* [ID:05]

Whilst participants generally did not respond well to penalties, these were noted as potentially effective at encouraging individual motivation. There was also a suggestion that a penalty deposit scheme could work with deprived communities, if the deposit was made on behalf of recipients.

*“I think it’s psychologically quite different if I give them that money and they lost it and got it, you know, this is developing their own internal incentive, and I like that a lot.”* [ID:11]

*“I think you could do it with deprived communities in that you could deposit an amount of money on their behalf and say you know, if you stick to it for this long you get so much of it …, and as time goes on ultimately they get all of it.”* [ID:05]

#### Form – cash versus vouchers

There was a common view that cash rewards were “more honest” than shopping voucher rewards and would allow individuals more freedom to use rewards as they chose. Despite this, cash was generally considered to be unacceptable in practice, because of the potential for “abuse”: particularly spending reward money on unhealthy products such as tobacco.

*“… what you wouldn’t want to give them is you know, £10, £12 to an individual because they’ve successfully lost weight only for them to potentially go and spend that money on a box of cigarettes…”* [ID:14]

There was also a view that vouchers would encourage recipients to “save up” rewards to purchase a larger item, rather than “fritter away” small amounts of cash.

*“… so if you’ve got sort of not hard cash but some other cash equivalent that you can save up to get something more meaningful, which was our experience of the women in the scheme.”* [ID:05]

#### Certainty - certain versus uncertain (lottery) rewards

Some participants felt that incentives should be certain – that is, that all potential recipients should receive a reward if they undertook the behaviour of interest – and not the result of a lottery for all those doing so. These individuals felt that uncertain rewards were unjust and potentially demotivating for recipients.

*“I just think if I was taking part in something that I’d been promised a reward if I do A and then actually I don’t get it because of, it’s only a chance, so someone else gets it, I’d feel that was really unjust and I’d feel cheated.”* [ID:05]

Others felt that as long as the chance of winning with a lottery-based HPFI were made transparent, such approaches were acceptable.

*“…so transparency I guess is really important and also the fact that they know the reward, the chance is there all of the time with lottery and/or they’re going to get a positive reward each time.”* [ID:14]

#### Magnitude

Overall, participants preferred smaller value rewards – although they were generally unable to articulate what a small value was. Larger rewards were often considered akin to bribery and as providing too much temptation to ‘game the system’. Reward value also raised issues of cost, cost-effectiveness and cost-saving discussed above.

*“Yeah, it would have to be a relatively low value for it to be acceptable.”* [ID:03]

*“I mean there are massive issues around costs at the moment … so the idea of using financial incentives in the time of austerity is probably something that we … aren’t going to get to look at … because at the moment, we’re looking at where we can make efficiencies.”* [ID:17]

#### Recipients – targeting, and individual versus groups

There was a general feeling that HPFI would be more acceptable if they were targeted at more vulnerable groups, particularly those living in deprived communities.

*“So I think it would be more acceptable for women from deprived communities … If it was something like that … it would just feel fairer.”* [ID:17]

*“It feels a little bit better because it’s a targeted intervention so it really is targeting the deprived community.”* [ID:03]

Group-based incentives were considered as useful in fostering peer support, but there was also a concern that this could lead to some individuals being alienated.

*“I quite like the reinforcing nature of that as kind of your reward being partly dependent on the behaviour of others as part of your team and that being a reinforcing measure.”* [ID:01]

*“I think that’s probably fraught with difficulty, so it could work well but equally you can see how the person who lets his or her behaviour slip is then seen as letting down the whole group and it could have all sorts of negative consequences.”* [ID:18]

## Discussion

This is the first study that we are aware of exploring the acceptability of HPFI to public health policymakers. Public health policymakers in our sample did not show universal or overwhelming support for HPFI, despite being provided with systematic review evidence supporting the effectiveness of HPFI. However, policymakers did acknowledge that HPFI could be a useful behaviour change tool. Areas of particular concern were doubt over the effectiveness and cost-effectiveness of HPFI, uncertainty about whether ‘gaming’ could be effectively and efficiently identified and prevented, and wariness that HPFI fail to address the underlying determinants of unhealthy behaviours. Participants also felt uneasy about the possibility that HPFI create and reinforce an expectation of instant rewards and discriminate against those who pursue healthy behaviours without financial rewards.

Public health policymakers identified a number of design elements that should be associated with more acceptable HPFI schemes. These included offering vouchers rather than cash, rewards rather than penalties, certain rather than lottery-based rewards, smaller value rewards (although these were not well defined), and incentives targeted at vulnerable groups – particularly those living in more deprived areas. Participants often seemed to second guess how other stakeholders, such as elected politicians, the public, and the media, would view HPFI and were particularly cautious of attracting negative responses from these stakeholders.

### Strengths and limitations of methods

Data saturation was achieved after around 19 interviews, indicating that the sample size was large enough. Qualitative research is not intended to be ‘generalisable’. Instead, validity is assessed in terms of triangulation and transferability of findings. As little previous research has explored acceptability of HPFI to policymakers, direct comparisons are not possible. However, as discussed below, findings were similar to other qualitative studies on the acceptability of HPFI to other groups [27, 32]. This increases the credibility of our findings.

We had clear research questions, structured our interview guide around these questions and report our results in relation to these research questions. Whilst this Framework Approach ensures that our results clearly reflect our aims, such an approach could be considered restrictive. We overcame this by using open coding to capture issues not initially anticipated.

### Interpretation of findings and comparison to previous results

Participants repeatedly stressed the need for new interventions to be cost-effective or even cost-saving. Other research has documented public concerns about the potential costs, and cost-effectiveness, of HPFI [13, 27, 32]. However, as far as we are aware, this is the first time cost-saving has been raised and this reflects the current climate of austerity and public sector cuts in English local government (where public health services are currently located). There is very little evidence concerning cost-effectiveness of HPFI. One recent randomised controlled trial of incentives of up to £400 (US$567) for smoking cessation during pregnancy reported a cost per quality-adjusted life-year gained of £482 (US$671) [33]. This figure is well below the current working maximum in England of £20–30,000 (US$28,779–43,168) per quality adjusted life-year gained [34], but does not reflect a cost-saving intervention (where the health-care savings achieved by an intervention outweigh the costs).

Although we provided participants with a summary of evidence from a recent systematic review on the effectiveness of HPFI [4], this appeared to have little influence on their views. This may because we introduced the evidence summary after we had invited participants to give their general reflections on HPFI: participants may have felt the evidence summary undermined the views they had already voiced. Alternatively, as peer-reviewed literature is only one type of ‘evidence’ that public health policymakers consider [35, 36], the other issues and concerns identified by participants may have been as, or more, important influences on their views than a systematic review. Some participants were critical of the existing research on HPFI – identifying selection bias and lack of long term follow-up as particular problems. This may explain their concerns about lack of effectiveness. Alternatively, underlying disquiet about HPFI may have led participants to be hyper-critical of research evidence.

As previously [13, 15, 27], participants were wary of the potential for ‘gaming’, where participants lie in order to gain rewards they are not entitled to. In trials of HPFI, little evidence of gaming has been documented [37]. It is, therefore, not clear if our participants’ concern was with preventing gaming itself, or with being seen to be preventing gaming. Previous authors have proposed restricting HPFI to behaviours that can be accurately measured in order both to prevent gaming and to show that this was being done [38].

A number of participants identified that HPFI do not address more distal, social, determinants of health and health behaviours and, as such, are unlikely to lead to sustained behaviour change. Other authors have expressed similar concerns that HPFI do not address the ‘root’ causes of unhealthy behaviours [12]. Whilst there is evidence that HPFI can have effects that last for at least 12 months after intervention withdrawal [4], longer term effects are not well documented. It is often suggested that HPFI act as external motivators that ‘crowd out’ internal motivation meaning effects are unlikely to be sustained once they are withdrawn [15]. Whilst ‘crowding out’ has been extensively reported in laboratory studies of economic behaviour [39], it does not appear to occur in relation to HPFI for health behaviours [40].

Our finding that policymakers prefer shopping vouchers to cash rewards reflects previous qualitative work with both members of the public and health care providers [22, 27, 41]. As previously, we found that participants were particularly concerned about the potential for recipients to use cash rewards to purchase unhealthy commodities such as tobacco and alcohol [27, 42, 43]. However, these findings contrast with those of two recent discrete choice experiments, that collected anonymous on-line data from members of the public, and found preferences for cash over voucher incentives [44, 45]. This discrepancy may be reflected in our participants’ recognition that, whilst recipients of HPFI would likely prefer cash, other stakeholders may not. Participants appeared to ‘second guess’ the preferences of other stakeholders, believing that vouchers were politically ‘safer’ than cash.

The preference for designing HPFI to encourage ‘saving up’, rather than ‘frittering away’, has been reported previously [46]. This may be linked to a conceptualisation of HPFI as serving dual purposes, particularly when targeted at disadvantaged populations: both as rewards, and as a tool to improve economic circumstances. Previous research has documented individual differences in how recipients choose to use HPFI rewards [46]. Clarity on what purpose HPFI should serve and how much recipients should be restricted in what they spend them on, may help in designing maximally acceptable HPFI.

Our finding of a strong preference for targeting HPFI at disadvantaged populations has been reported previously in a qualitative study with members of the public [27] and may reflect a perception that HPFI may be most effective in more disadvantaged people [38]. Whilst this makes intuitive sense, there is very limited evidence concerning differential effectiveness of HPFI by socio-economic status [4]. Furthermore, it is not a universal finding that targeted HPFI are preferred [32, 44]. An alternative explanation of the preference for targeted schemes is that these may be considered both cheaper (as fewer people are eligible for rewards), and more cost-effective (because a higher proportion of people may respond).

Ultimately our interviews with policymakers identified three areas of tension. The first was a tension between wanting to trust individuals, and designing incentive schemes that promote individual accountability and responsibility; and an inherent distrust of individuals with concerns around individuals gaming the system. Secondly, there was a tension between participants’ own views; and concerns about the possible views of other stakeholders such as the public, media and politicians. Thirdly, there was a tension between personal and professional views on incentives; policymakers sometimes suggested a distaste for HPFI on a personal level, but could see the value of them from a professional point of view.

### Implications for policy, practice and research

Overall, we found that policymakers raised similar concerns about HPFI as other stakeholder groups [13, 15, 27]. However, policymakers appear to be unique in explicitly considering the possible views of other stakeholders when considering the acceptability of HPFI.

Our results suggest that HPFI would be more acceptable to UK policymakers if there was further evidence of long-term effectiveness and cost-effectiveness, and robust strategies to explicitly minimise gaming. Schemes that provided shopping voucher rewards, of smaller value, particularly targeted at deprived group would be most acceptable to policymakers.

Whilst there is limited evidence on the cost-effectiveness of HPFI, there are now a number of systematic, and other, reviews of effectiveness [3, 4, 6, 7, 25]. Researchers should focus their efforts on establishing cost-effectiveness and communicating results to policymakers.

Given participants’ concerns with others’ views on HPFI, there may be a need for further qualitative work to uncover the views of these groups. Research suggests that media coverage of HPFI in the UK is generally overall balanced, or favourable [47], and policymakers could be reassured of this. Effective communication of the results of existing research on acceptability of HPFI may help policymakers understand key areas of concern and how these could be overcome.

## Conclusions

Public health policymakers working in England acknowledged that HPFI could be useful behaviour change tools, but were not overwhelmingly supportive of these interventions. They raised particular concerns about effectiveness and cost-effectiveness, potential ‘gaming’, and whether or not HPFI address the underlying causes of unhealthy behaviours. Shopping voucher rewards, of smaller value, targeted at deprived groups were most acceptable.

## Appendix 1 - Interview topic guide

Introductions, information, questions, & consent

Initial responses to generic concept of financial incentives for healthy behaviours

Reaction to information on effectiveness of financial incentives for healthy behaviours

Specific examples of financial incentives for healthy behaviours

- Example 1: breastfeeding
- Example 2: smoking in pregnancy
- Example 3: weight loss

Aspects of design of financial incentives for health behaviours

Close and thank you

## Appendix 2 - Text of show cards

### Show card 1

A definition of financial incentives: “cash or cash-like rewards or penalties provided to, or imposed on, individuals contingent on their performance of healthy behaviours”

### Show card 2

Recent systematic review evidence: “We conducted a systematic review of controlled evaluations of the effectiveness of financial incentive interventions, compared to no intervention or usual care, to encourage healthy behaviour change, in non-clinical adult populations, living in high-income countries. On average, incentives were 2.5 times more effective than usual care for smoking cessation in the short term (<6 months) and 1.5 times as effective in the longer term. Financial incentives were 1.9 times more effective than usual care for encouraging people to attend for vaccination or screening. Overall, incentives were 1.6 as effective as usual care in encouraging uptake of healthy behaviours.”

### Show card 3

Example 1, breastfeeding: “A local authority in Yorkshire has low breastfeeding initiation and maintenance rates. New mothers are offered £200 in cash if they are still doing any breastfeeding at 6 months. Peer supporters continue to provide normal health, advice and support. This example is based on a pilot scheme being evaluated by researchers at the University of Sheffield.”

### Show card 4

Example 2, smoking in pregnancy: “A region in Scotland has high rates of smoking in deprived, pregnant women. Pregnant women are offered cessation support from community pharmacies. They set a quit date and then return weekly for support over 12 weeks. Each week that they return and provide a smoke-free breath test they are rewarded with £12.50 in supermarket vouchers. This example is based on the ‘Give it up for baby’ programme in Tayside, Scotland.”

### Show card 5

Example 3, weight loss: “A local authority on the south coast of England has high overweight and obesity rates. They commission an online weight loss and maintenance service that provides weight loss resources and incentives. Participants are provided with an individualised health weight loss plan and supervised weigh-ins take place monthly. Participants deposit a lump sum at the start of the programme and receive a proportion of this back each month for every pound that they lose – up to a maximum of £425 over one year. This example is based on the ‘Pounds for pounds’ programme in Kent.”

### Show card 6

1. Reward or penalty/deposit contract
2. Cash or shopping vouchers or specific ‘prizes’
3. Total value
4. Everyone eligible receives reward/penalty, or lottery to determine who receives
5. Reward/penalty for doing something that should help people adopt healthier behaviours (e.g. attending a health promotion session) or for actually adopting healthier behaviours (e.g. taking more steps per day)
6. All instances of healthy behaviours rewarded/penalised or only some instances
7. Fixed reward/penalty or escalating schedule – the longer you stick to the programme, the higher the reward
8. Reward/penalty given to individuals or groups of individuals working together
